# Free-breathing non-contrast MRA with efficiency-adaptive self navigation

**DOI:** 10.1186/1532-429X-15-S1-P233

**Published:** 2013-01-30

**Authors:** Yibin Xie, Zhaoyang Fan, Rola Saouaf, Yutaka Natsuaki, Gerhard Laub, Debiao Li

**Affiliations:** 1 Cedars Sinai Medical Center, Los Angeles, CA, USA; 2 University of California, Los Angeles, Los Angeles, CA, USA; 3 Siemens Healthcare, Los Angeles, CA, USA

## Background

Non-contrast MRA (NC-MRA) based on bSSFP and slab-selective inversion has become an attractive alternative for imaging renal arteries without the usage of potentially nephrotoxic contrast agent. It typically requires navigator gating or abdominal bellow triggering to alleviate breathing motion artifacts. However, navigator gating significantly complicates and lengthens exams due to its setup, adjusting, and scout scans. It also causes signal loss in renal arteries due to cross-pair saturation bands. Abdominal bellow triggering increases patient preparation time and disables the usage of ECG triggering leading to suboptimal inflow effect. In this work, a novel self navigation (SN) technique is developed in an attempt to overcome the limitations of the aforementioned free-breathing methods while maintaining scanning efficiency.

## Methods

**Figure 1:**
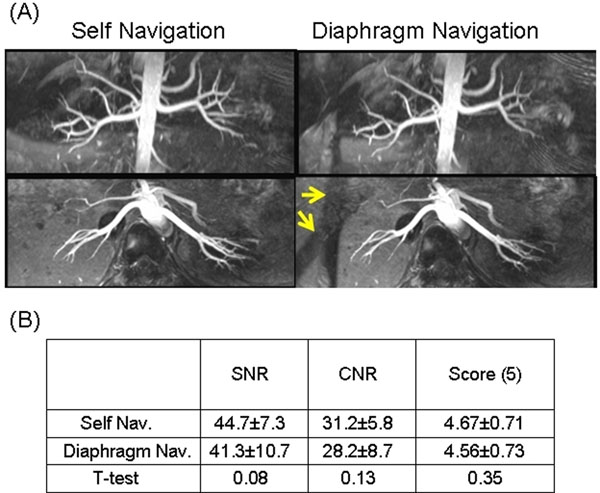
(A) MIP images of self-navigated (left column) and conventional (right column) renal NC-MRA showing similar image quality. Yellow arrows point to saturation bands caused by conventional navigators. (B) Image quality comparison statistics.

An SN readout line modified to superior-inferior (SI) direction without partition or phase encoding is inserted at the end of each 3D bSSFP readout block (Fig. 1A). Fourier transform of the SN readout line is the 1D projection of the entire imaging slab in the SI direction, which serves as the ‘fingerprint' of the current respiratory phase. The reference projection profile is defined in the first two repetitions in the scan with breath-hold. In each subsequent repetition, the correlation coefficient (CC) is calculated between current projection profile and the reference profile. Respiratory motion is detected if the CC value drops below the threshold and current image lines will be rejected and reacquired in the next repetition. The threshold is dynamically adapted to maintain scanning efficiency.

Nine healthy volunteers with IRB approval were scanned on a 3T clinical scanner (MAGNETOM Verio, Siemens) with the following scan parameters: repetition = 700-900 ms; TI = 550-750 ms; acquisition time = 4-6 min depending on subject heart rate; TE/TR = 1.9/3.8 ms; 3D transverse slab with left-right readout; FOV = 400x250 mm2, matrix = 304x192, slice thickness = 2.2 (1.1 interpolated) mm, yielding isotropic resolution = 1.1 mm3; iPAT = 2; bandwidth = 780 Hz/pixel; FA = 90. For comparison, conventional navigator gated bSSFP MRA images were acquired immediately afterwards using same parameters.

## Results

**Figure 2:**
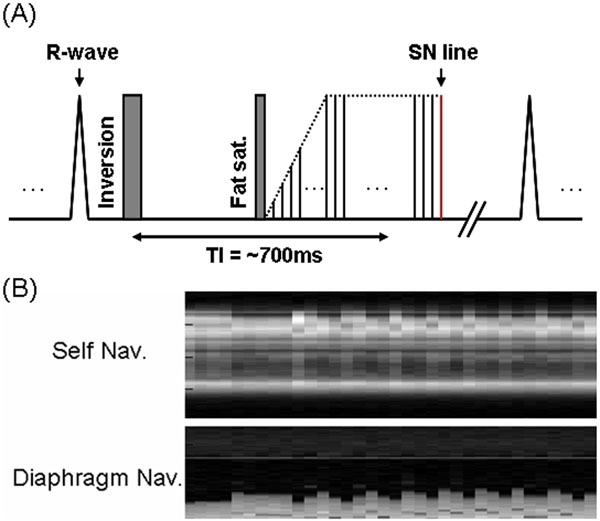
(A) Pulse diagram of SN NC-MRA. (B) Representative SN profiles (top) and diaphragm Navigator signal (bottom) showing high agreement.

SN projections clearly show the underlining respiratory motion and are highly matched with diaphragm navigator (Fig. 1B). Excellent depiction of the intra- and extrarenal arteries are achieved using SN with no navigator saturation bands (Fig. 2A). No statistically significant difference was found between the two gating methods regarding SNR and CNR, as well as qualitative reviewer scores (Figure. 2B).

## Conclusions

Preliminary results of SN bSSFP NC-MRA have demonstrated comparable image quality to conventional navigator gated acquisition but much simplified imaging planning and absence of saturation bands. Its performance in patients is currently under investigation.

## Funding

This work is funded by grant NIH/NHLBI R01HL096119

